# Cost-effective Whole Exome Sequencing discovers pathogenic variant causing Neurofibromatosis type 1 in a family from Jammu and Kashmir, India

**DOI:** 10.1038/s41598-023-34941-y

**Published:** 2023-05-15

**Authors:** Akshi Spolia, Arshia Angural, Varun Sharma, Shipra, Sushil Razdan, Manoj K. Dhar, Ankit Mahajan, Vijeshwar Verma, Kamal K. Pandita, Swarkar Sharma, Ekta Rai

**Affiliations:** 1 Human Genetics Research Group, School of Biotechnology, Shri Mata Vaishno Devi University, Kakryal, Jammu and Kashmir, 182320 India; 2 Bhagwati Nagar, House No.:7, 180016 Jammu and Kashmir, India; 3 School of Biotechnology, University of Jammu, Jammu and Kashmir, 180006 India; 4 Health Clinic, Swarn Vihar, Muthi, 181205 Jammu and Kashmir, India; 5 Present Address: Department of Medical Genetics, JSS Medical College and JSS Hospital, JSS Academy of Higher Education and Research, Mysuru, Karnataka 570015 India; 6 Present Address: NMC Genetics India Pvt Ltd, Gurugram, 122002 Haryana India; 7 Present Address: Centre for Molecular Biology, Central University of Jammu, Jammu and Kashmir, 181143 India

**Keywords:** Biotechnology, Computational biology and bioinformatics, Genetics, Molecular biology, Neuroscience, Diseases, Medical research, Neurology

## Abstract

Neurofibromatosis type 1 (NF1) is a multisystemic hereditary disorder associated with an increased risk of benign and malignant tumor formation predominantly on the skin, bone, and peripheral nervous system. It has been reported that out of all the NF1 cases, more than 95% cases develop the disease due to heterozygous loss-of-function variants in Neurofibromin (*NF1*) gene. However, identification of *NF1* causative variants by presently recommended method of gene-targeted Sanger sequencing is challenging and cost-intensive due to the large size of the *NF1*gene with 60 exons spanning about 350 kb. Further, conducting the genetic studies is difficult in low resource regions and among families with the limited financial capabilities, restricting them from availing diagnostic as well as proper disease management measures. Here, we studied a three-generation family from Jammu and Kashmir state in India, with multiple affected family members showing clinical indications of NF1. We combinedly used two applications, Whole Exome Sequencing (WES) and Sanger sequencing, for this study and discovered a nonsense variant NM_000267.3:c.2041C>T (NP_000258.1:p.Arg681Ter*) in exon 18 of *NF1* gene in a cost effective manner. In silico analyses further substantiated the pathogenicity of this novel variant. The study also emphasized on the role of Next Generation Sequencing (NGS) as a cost-effective method for the discovery of pathogenic variants in disorders with known phenotypes found in large sized candidate genes. The current study is the first study based on the genetic characterization of NF1 from Jammu and Kashmir–India, highlighting the importance of the described methodology adopted for the identification and understanding of the disease in low resource region. The early diagnosis of genetic disorders would open the door to appropriate genetic counseling, reducing the disease burden in the affected families and the general population at large.

## Introduction

Neurofibromatosis type 1 (NF1) (OMIM#162200) is a multisystem autosomal dominant disorder that primarily occurs due to underpinning pathogenic variants in the Neurofibromin gene (*NF1*;OMIM# 613113) cytogenetically located on chromosome 17q11.2^1^. In published literature, its incidence is reported as 1 per 2500 to 3000 individuals^2^. However, incidental reports on NF1 in the Indian populations are unavailable. A positive family history has been observed among only 50% of the reported NF1 cases; however, the rest are caused by spontaneous variants in *NF1* gene^3^. The clinical hallmark characteristics of NF1 are presence of café-au-lait colored spots (six or more) in early age, multiple neurofibromas, grion or axillary freckles, gliomain optic nerves, yellowish-brown solid dome-shaped lesions (Lisch nodules) over the surface of iris^4^. Neurofibromas are benign tumors of the peripheral nerve sheath, with a further categorization of plexiform, cutaneous, and subcutaneous types^5^. NF1-associated gliomas can develop in various parts of the brain, however the majority of them occur in the optic chiasm, nerve sand it’s tracks^6^.

Despite the known fact that pathogenic *NF1* variants cause the disease, most of the NF1 patients and their families in the J&K region of India prefer not to undergo any genetic evaluation due to their limited financial capabilities. Further, a delay in the clinical confirmation of the disease due to limited diagnostic resources in this region, deprive them from timely acquiring the facilities of therapeutic management. Given the lack of timely genetic counseling and management interventions to the affected ones, the disease burden in families and populations becomes huge over time.

**Figure 1:**
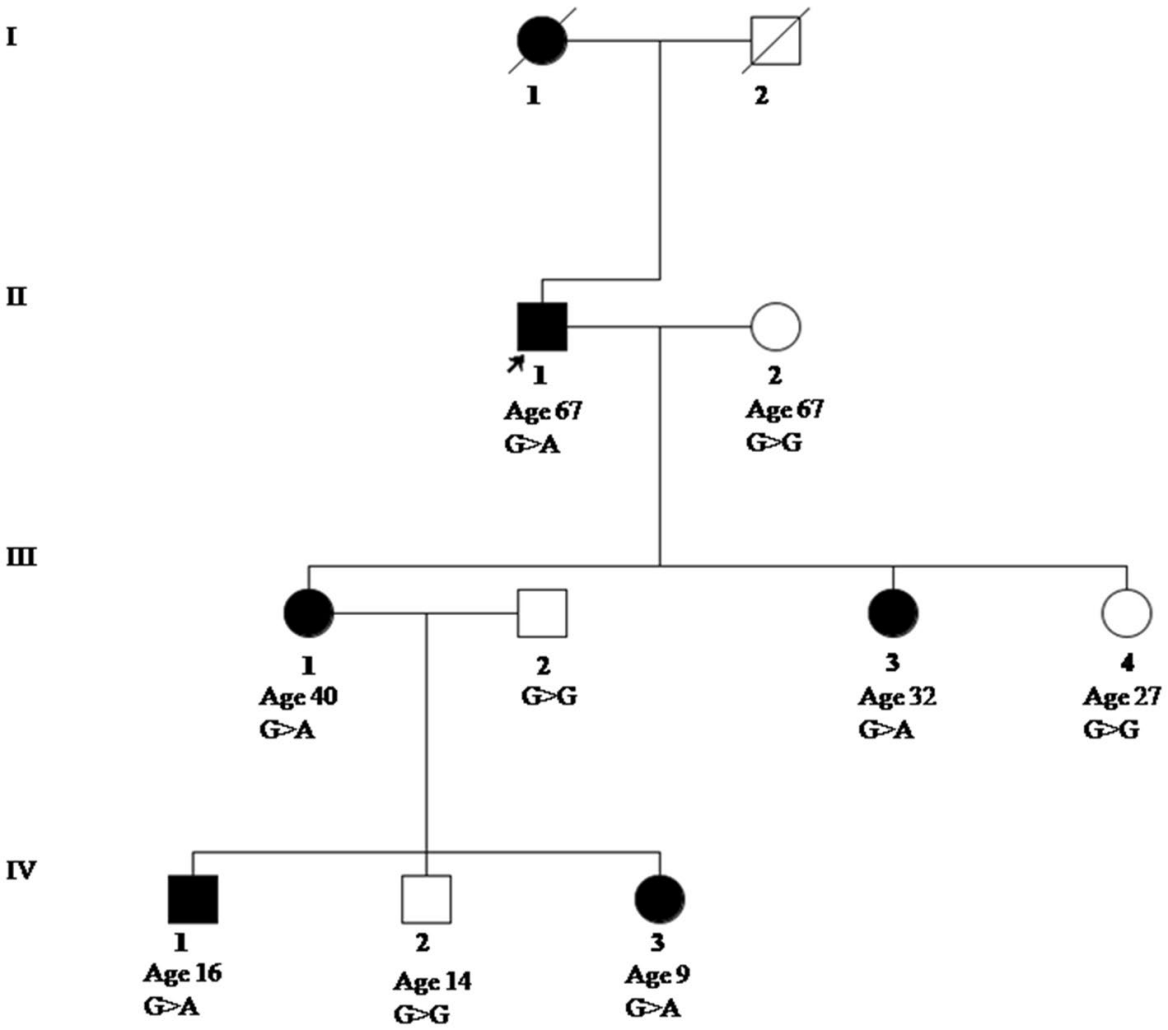
Pedigree of NF1 family representing clinical status of the proband II(1) and his family along with their genotypes and age. Males are represented in square and females in circle (affected with solid representations). The proband II(1) has been marked with a black arrow.

In this study, we present a three-generation family with multiple affected members having clinical features corresponding to NF1 (Fig. 1). The family was genetically tested for the variants in disease-associated *NF1* gene. A nonsense variant NM_000267.3:c.2041C>T (NP_000258.1:p.Arg681Ter*) in exon 18 of *NF1* gene was identified in the affected individuals through targeted Whole-Exome sequencing initially and then confirmed via sanger sequencing. Screening of variant in other family members showed its segregation with the disease phenotype in an autosomal dominant manner. This study highlights the role of Next Generation Sequencing (NGS) as a cost-effective method for the diagnosis of rare genetic disorders and identification of known and new causal variants, especially for large size gene in potential candidate regions.

## Clinical presentation of the disease in the family

A family from Jammu and Kashmir region of India was recruited presenting clinical features suggestive of Neurofibromatosis type1 (Supplementary Fig. 2a,b). The proband II(1), a 67 year old male, presented with clinical features such as multiple soft tissue cutaneous nodules (neurofibroma) of different sizes all over the body. II(1) was suffering from type 2 diabetes and underwent bypass heart surgery. Of his three offspring (all daughters), two 40 years old III(1)and 32 years old III(3) were suffering from NF1, whereas the youngest who was 25 years old III(4) was unaffected. Both the affected daughters III(1) and III(3) had multiple cutaneous neurofibromas and multiple hyperpigmented macules (Café-au-lait pigmentation), however, the symptoms were more severe in the elder one III(1). Furthermore, III(1) had three offspring of which two were affected [16 years old son IV(1) and 9 years old daughter IV(3)] and one was unaffected IV(2). The affected son IV(1) had few cutaneous neurofibromas and hyperpigmented macules (Café-au-lait pigmentation), whereas the affected daughter IV(3) had few hyperpigmented macules (Café-au-lait pigmentation) as well as poor intellectual skills. She had a history of neurological glioma and ophthalmological abnormalities. At the age of 8 years, IV(3) experienced a regular single episode of vomiting per day associated with gait disturbances for which she was diagnosed with right cerebellar Pilocytic astrocytoma (grade 1). On the basis of her radiological findings, IV(3) underwent a craniotomy for the decompression of a tumor that was causing midline suboccipital compression. Post-surgery, she experienced diminution of vision because of bilateral papilledema for which she was operated for the second time.

Despite the fact that the clinical findings were highly suggestive of NF1, the family could not timely understand the hereditary nature of the disease and did not undergo any genetic investigation. Had it been diagnosed earlier, when the proband or his daughters presented NF1 manifestations, the family could have undergone relevant screening and counseling programmes and understood the autosomal dominant nature of the disease; but due to lack of information and resources in the region, there was no timely intervention and genetic counseling, resulting in an continued trans-generational burden of NF1 disease in the family (as seen in the third generation).

## Materials and methods

### Sample collection and DNA extraction

After a thorough clinical, phenotypic evaluation of the proband II(1) and other family members including both affected [II(1), III(1), and IV(3)] as well as unaffected [III(4), and IV(2)], approximately 2 mL of blood was collected with their informed consents. The informed consents have been obtained from all the subjects under study, also ethical approval for the study was provided by the Institutional Ethical Review Committee (IERB) [under IERB Serial No: SMVDU/IERB/18/67] of Shri Mata Vaishno Devi University (SMVDU), and all experiments were performed in accordance with its guidelines and regulations. For DNA extraction, collected blood samples were processed using standard protocols of FlexiGene® DNA isolation kit (QIAGEN, AGKT-FG-64). Quality check and DNA quantification of the isolated samples was performed using agarose gel electrophoresis (0.8% agarose) and BioSpectrometer®, respectively.

### Whole exome sequencing (WES)

Keeping in mind the limited financial resources, and fact that NF1 gene, a very large gene, is the most plausible candidate gene, the DNA sample of the proband II(1) was subjected to Next Genome Sequencing. However, instead of targeted gene sequencing for NF1, Whole Exome Sequencing (WES) was opted in anticipation of additional data, in case NF1 gene does not show casual variant. WES was carried out with a complementary support from MGI Tech Co., Ltd. MGI Easy Exome Universal Library Prep SetV1.0 with the MGI Easy Exome Capture V5 Probe Set (MGI Tech Co., Ltd., China) was used for DNA library preparation and target enrichment strictly following the manufacturer’s protocols. Paired-end sequencing of single-indexed adapter-ligated Whole-Exome enriched DNA library was performed on MGISEQ-2000RS sequencing instrument, resulting in a yield of 150-bp paired-end reads through High-throughput Sequencing Set (PE150). Base calling was performed with *ZebracallV2* software (MGI Tech Co., Ltd.) which is integrated into instrument control software of MGISEQ-2000RS (MGI Tech Co., Ltd.) and raw Fastq files have been generated following demultiplex of sequencing data done by instrument control software of MGISEQ-2000RS, which were used for analysis.

### Raw data analysis

The raw Fastq sequencing data generated from the WES library was further processed through Varstation (https://varstation.com/en/, date of access: 12-02-2021). It is an online cloud-based tool of pipelines for NGS data analysis and variant identification/annotation/interpretation in human genome. It consists of three main steps of genome analysis based on Bioinformatics pipelines alignment with the sequence reads of the reference genome; variant calling and annotation^7^. The major steps of the Varstation pipeline used for this data analysis are described below:

- *Reads pre-processing* The quality control (QC) of the raw reads in the .fastq file and their pre-processing was performed using FastQC version 2^8^, BEDTools version 2.18^9^, BamTools version 2.5.2^10^ and VCFtools version 4.1^11^, after the adapter trimming using Cutadapt.
- *Alignment of reads* After quality control check of raw data and its pre-processing, the generated clean reads were aligned and mapped to the reference human genome assembly GRCh37/hg19using two tools: Burrows–Wheeler Aligner (BWA) version 0.7017 (r1188)^12^ and Torrent Mapping Alignment Program (TMAP) version 5.16.0 (GitHub: https://github.com/iontorrent/TS/tree/master/Analysis/TMAP, date of access:03-03-2021).
- *Post-processing of aligned reads* PCR duplicates were removed using the Picard tools version 2.26.3 (https://broadinstitute.github.io/picard/).
- *Variant calling* Identification of variants from sequence data was performed through variant calling tools: GATK version^13^ 1.2.1.0 –Unified Genotyper and Haplotype Caller, SAM tools version 1.9^14^, FreeBayes version 1.3.6^15^, Atlas version 0.9.9^16^ and smCounter version 2^17^.
- *Variant annotation and filtration* The variant annotations were performed using the annotation algorithm ANNOVAR version 0.11.9^18^.

A total of 56,829 variants were annotated in the data. We prioritized analyses of variants present in NF1 gene located on chromosome 17. Variant confirmation was done by manual variant filtration and prioritization, following the guidelines recommended by the American College of Medical Genetics and Genomics (the ACMG guidelines)^19^. Variant observed was further validated by manual visualization on Integrative Genomics Viewer (IGV, https://igv.org/)^20^.

### Variant confirmation using Sanger sequencing

The variant identified using the WES approach was confirmed in proband II(1), other affected as well as unaffected family members using targeted Sanger sequencing. Polymerase chain reaction (PCR) was carried out to amplify *NF1* exon18 along with its exon/intron boundaries. Electropherograms were analyzed through Chromas v2.5.1(http://technelysium.com.au, date of access: 15-03-2021) and Sequence Scanner Software v2.0 (http://sequence-scanner-software.software.informer.com, date of access: 24-05-2021), using genomic DNA reference sequence NM_000267 GRCh37/hg19 Assembly. The pathogenicity of the identified variant was confirmed using various online prediction tools such as Mutation Taster 2 (http://mutationtaster.org/, date of access: 10-06-2021); Combined Annotation-Dependent Depletion (CADD) version 1.1^21^; and Mendelian Clinically Applicable Pathogenicity (M-CAP) version 1.3^22^.

### Ethical approval

The study was approved by the Institutional Ethical Review Committee (IERB), SMVDU, J&K, India, under IERB Serial No: SMVDU/IERB/18/67. All relevant ethical guidelines have been followed, all necessary IERB and/or ethics committee approvals have been obtained, also all necessary patient/participant consent has been obtained and the appropriate institutional forms have been archived. The study was not carried with any financial support from the recruited family.

### Informed consent

Informed consent was obtained from all individual participants included in the study. For minor patients, consent has been taken from the parents.

## Results and discussion

Analysis of variants in NF1 gene from the WES data of proband II(1) showed 18 variants in gene, of which 15 variations were intronic and 3 exonic variations, of which two of the exonic variations were synonymous. The analyses revealed a non-sense NM_000267.3:c.2041C>T (NP_000258.1:p.Arg681Ter*) variation, located in exon18 of *NF1*gene. This variant is already listed in the National Institutes of Health (NIH), USA ClinVar database (https://www.ncbi.nlm.nih.gov/clinvar/, date of access: 25-08-2021) with Variation ID:188280 and associated with Neurofibromatosis type 1^23–27^. Validation by Sanger sequencing in the proband II(1), as well as the rest of the family members, both affected and unaffected, has revealed its segregation in an autosomal dominant pattern. The electropherograms marked with change at nucleotide position on the reverse strand are shown in Supplementary Fig. 1. The proband II(1) and other affected individuals, III(1) and IV(3) are heterozygous for the identified variant, whereas unaffected individuals III(4) and IV(2) are homozygous for the wild type. This identified variant (NM_000267.3:c.2041C>T) has been submitted in ClinVar database with NF1 ClinVar accession number: SCV001762282. This variant causes an early codon termination resulting in a truncated mRNA and protein, vulnerable to nonsense-mediated decay^28^. The pathogenicity prediction tools also predicted pathogenic nature of this variant (Mutation Taster, p-score = 1.00; Combined Annotation Dependent Depletion (CADD) Phred score (Threshold > 20) = 39; Mendelian Clinically Applicable Pathogenicity (M-CAP) score (Threshold > 0.025) = 0.298). Also, the c.2041C>T stop-gain variant in the *NF1* gene was found to be highly conserved throughout most of the mammalian species. Previous functional studies confirm that this variant significantly reduces the expression and function of NF1 protein as well as increases ERK (extracellular signal-regulated kinases) levels^29^ and thus, is known to be pathogenic^30^. A study onNf1 ± designed mouse model shows that this nonsense variant, (p.R681X), results in the truncation of neurofibromin which is composed of 680 amino acids and causes the development of ocular gliomas with enlarged optic nerve volumes^31,32^. Previously reported NF1 patients harboring the R681X mutation are reported to present with a range of clinical symptoms, such as cutaneous neurofibromas^33^, learning disability^34^, optic glioma and precocious puberty^31^, etc.

Molecular confirmation of NF1 disease is difficult, especially in low resource regions, due to the large size of the gene, lack of information on other mutational hotspots and a complicated *NF1* mutational spectrum. *NF1* variants have been screened through various other techniques such as real-time PCR-based gene dosage (large deletion confirmation), intragenic *NF1* microsatellite analysis (for large deletion screening); and *NF1* Sanger sequencing at both cDNA (point mutation screening), and DNA levels (point mutation confirmation)^35^. When a molecular abnormality is not identified at the cDNA level, exon deletion/duplication screening is usually being performed using Multiplex Ligation-dependent Probe Amplification (MLPA) method. But these procedures are usually time-consuming, expensive and labor intensive^36^. Therefore, NGS represents a significant advancement in *NF1* gene molecular screening, being more cost-effective and throughput-efficient as compared to other techniques^36^. The present study highlights its importance particularly in regions with limited resources.

Since the *NF1* gene expresses in an autosomal dominant manner, only one of the two copies of the gene must be altered in order to manifest the disorder. Hence, there is a one in two probability that an offspring may acquire the disorder from just one affected parents^37^. Although around 50% of cases are caused by novel variants that emerge as a de novo lesion in the *NF1* gene^38^. Among all NF1 cases, more than 95% are due to heterozygous loss-of-function variant in the *NF1* gene, with point mutations accounting for roughly 90% of cases and bigger deletions of about 1–1.5 MB accounting for roughly 10% of cases^39^. In approximately 80% of NF1 cases, genetic variations lead to the generation of premature termination codons (PTCs), resulting in the synthesis of a truncated neurofibromin^40^ and mouse models have indicated in past that premature termination act as a null allele resulting in a complete loss of NF1 function^29^. Also, previously reported studies have shown termination variation results in NF1-OPG (Optic pathway Glioma)^32^. In this study too, one of the family members, that is heterozygous IV(3), shows complications associated with OPG, including Pilocytic astrocytoma (grade1) and bilateral optic disc edema due to bilateral papilledema/anterior ischemic optic neuropathy (AION), causing vision loss. The OPGs are the most common type of brain tumor associated with the NF1, affecting about 15–20% of NF1 patients^39^. It accounts for about 5% of low-grade gliomas in children and about 70% are present in children with NF1. Also, optic disc edema due to bilateral papilledema related with Neurofibromatosis type 1 was reported in previous case study^41^. Clinically, NF1 is monitored without any medicinal or surgical procedures that would be different from those utilized in the general population for similar signs and symptoms. Surgical removal of cutaneous neurofibromas that affect quality of life is possible, but recurrence is quite common. Patients with large painful plexiform neurofibromas with high progression are suitable for surgical treatment. Patients with a lower quality of life resulting from cosmetic defects often seek laser treatment for the spots and freckles, keeping in mind that recurrence is common^42^. When radiological and clinical data indicates disease development, carboplatin and vincristine are the prime treatments for NF1-related OPGs^43^.

## Conclusion and future perspective

In conclusion, we provide the first confirmed report of Neurofibromatosis type 1 (NF1) from Jammu and Kashmir region of India associated with an already known disease-causing variant NM_000267.3:c.2041C>T, and its association with phenotypic features of OPG (optic pathway glioma) in one of the NF1 affected member IV(3). At this juncture, it is recommended that adoption of genetic counselling and pre-natal diagnostic measures may help the family with respect to the future generations. Further, as the burden of genetic disorders in the population of Jammu and Kashmir appears to be high due to several factors including strict caste endogamy, practice of consanguinity, lack of awareness on genetic disorders and limited resources, a population level screening program in this region is highly required^44^. It is anticipated that initiatives to catalog genetic variants (along with potential pathological inferences) may help create awareness in the population and lead to better management of rare disorders, including NF1. Further, we propose adoption of Next Generation Sequencing for the discovery of disease-causing variations in disorders associated with large sized candidate genes, considering local financial constraints as well as the efficiency afforded by bringing timely diagnosis and management opportunities to suspected families in low resource regions.

## Supplementary Information

Supplementary Information.

## Data Availability

The variant and data in the current study is available in the ClinVar database repository with ClinVar accession number: SCV001762282.1. [https://www.ncbi.nlm.nih.gov/clinvar/submitters/505856/], [https://www.ncbi.nlm.nih.gov/clinvar/variation/VCV000188280.32].
