# Supplementary material for: Cost-effective Whole Exome Sequencing discovers pathogenic variant causing Neurofibromatosis type 1 in a family from Jammu and Kashmir, India
Source: Sci Rep. 2023 May 15;13:7852. doi: 10.1038/s41598-023-34941-y (PMC10185681; doi:10.1038/s41598-023-34941-y)
Supplement: Supplementary file 1 — Supplementary Information. [file 41598_2023_34941_MOESM1_ESM.docx]

Supplementary Figure 1


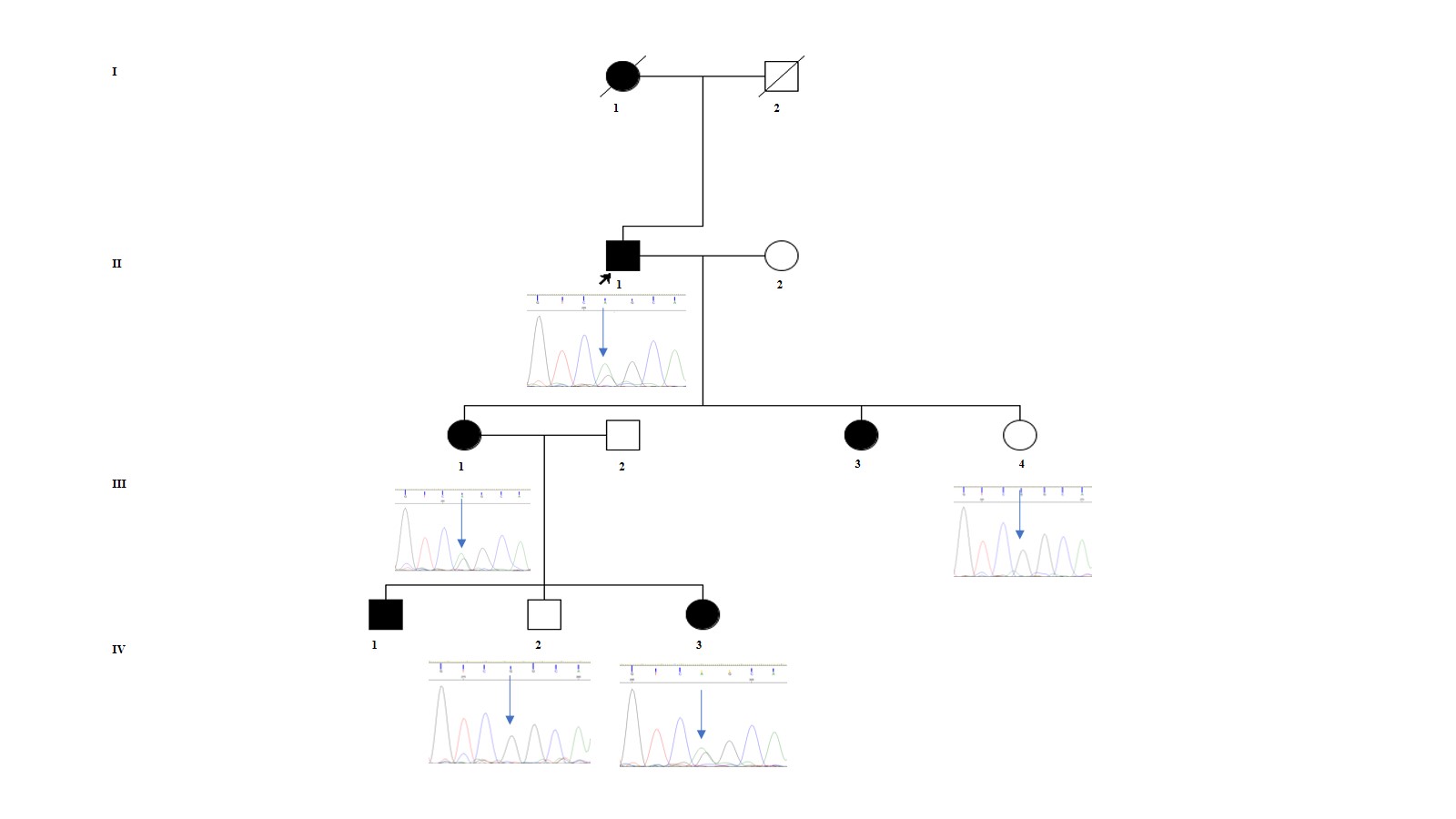


**Supplementary Figure 1.** Represents pedigree of the recruited NF1 family along with their representative screenshots of the Sanger Sequencing electropherograms of the identified variant NM_000267.3:c.2041C>T (reverse strand sequencing data G>A) confirming autosomal mode of inheritance. An arrow on the electropherogram indicates stop-gain variation in exon18 of NF1 gene. The proband II(1) and other affected individuals III(1) and IV(3) were found to be heterozygous (G>A) for the variation, whereas unaffected III(4) and IV(2) was found to be homozygous (G>G).

**Supplementary Figure 2**

**a b**

**
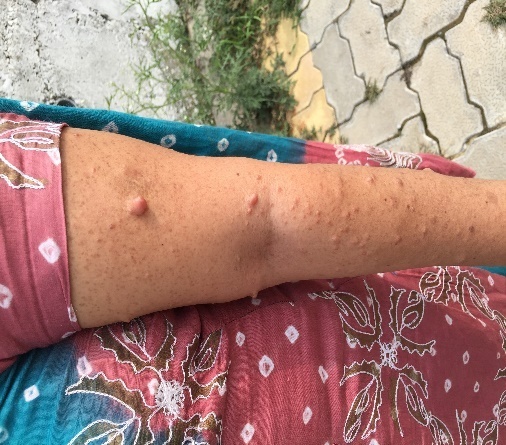

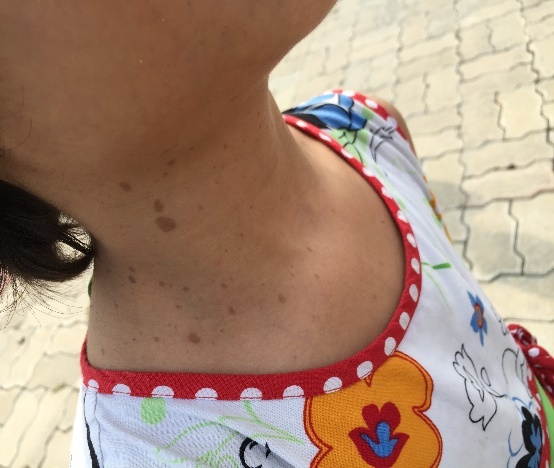
**

**Supplementary Figure 2. a** Represents zoomed image of multiple hyperpigmented macules (Café-au-lait pigmentation), **b** Represents zoomed image of neurofibroma and multiple hyperpigmented macules (Café-au-lait pigmentation) in the affected individuals.

Supplementary Figure 3

**Supplementary Figure 3.** Variation filteration stratergy applied in II(1) WES data.
